# Incidence of anterior uveitis in patients with axial spondyloarthritis treated with anti-TNF or anti-IL17A: a systematic review, a pairwise and network meta-analysis of randomized controlled trials

**DOI:** 10.1186/s13075-021-02549-0

**Published:** 2021-07-16

**Authors:** Damien Roche, Martin Badard, Laurent Boyer, Pierre Lafforgue, Thao Pham

**Affiliations:** 1 APHM, CHU Sainte-Marguerite, Rheumatology Department, Aix Marseille Univ., Marseille, France; 2 Rheumatology Department, Saint Joseph Hospital, 26 Boulevard de Louvain, 13285 Marseille, France; 3 School of Medicine, La Timone Medical Campus, EA 3279: CEReSS - Health Service Research and Quality of life Center, Aix Marseille Univ., Marseille, France

**Keywords:** Axial spondyloarthritis, Meta-analysis, Anti-TNF, Anti-IL17A, Uveitis

## Abstract

**Background:**

Anterior uveitis (AU) is the most frequent extra-articular feature of axial spondyloarthritis (axSpA). We aimed to assess and compare the incidence of AU in axSpA patients treated with anti-TNF or anti-IL17A.

**Methods:**

We systematically reviewed PubMed, EMBase, and Cochrane from inception to May 3, 2020, and searched for placebo-controlled and head-to-head randomized controlled trials (RCTs) assessing anti-TNF monoclonal antibodies (mAb) or soluble receptor fusion protein or anti-IL17A in patients with axSpA according to ASAS criteria and reporting safety data on AU. Data were extracted following a predefined protocol. We did pairwise and network meta-analyses for the primary outcome of AU flares (relapse or de novo) incidence and estimated summary odds ratios (ORs). We assessed the quality of evidence using the Cochrane risk-of-bias 2.0 tool. We ranked treatments according to their effectiveness in preventing AU flare using the P-score.

**Results:**

We identified 752 citations and included 33 RCTs, comprising 4544 treated patients (anti-TNF mAb 2101, etanercept [ETN] 699, anti-IL17A 1744) and 2497 placebo-receiving patients. Incidence of uveitis was lower with anti-TNF mAb versus placebo (OR = 0.46; CI 95% [0.24; 0.90]) and versus anti-IL17A (OR = 0.34; CI 95% [0.12; 0.92]. According to the P-score, the ranking from the most to the least preventive treatment of uveitis flare was as follows: anti-TNF mAb, ETN, placebo, and anti-IL17A.

**Conclusion:**

In RCTs assessing anti-TNF and anti-IL17A in axSpA, incident uveitis are rare events. However, this network meta-analysis demonstrates that anti-TNF mAb are associated with a lower incidence of uveitis compared to placebo and anti-IL17A.

**Supplementary Information:**

The online version contains supplementary material available at 10.1186/s13075-021-02549-0.

## Background

Axial spondyloarthritis [axSpA] is a chronic inflammatory disease that mainly affects the axial skeleton. AxSpA can be classified into two subgroups: ankylosing spondylitis [AS] and non-radiographic axSpA [nr-axSpA] [1, 2]. The most frequent extra-articular manifestation associated with axSpA is acute anterior uveitis [AAU] which occurs in 23% – 33% of patients with AS [3, 4], and in 16% of patients with nr-axSpA [3].

Biologics therapy has dramatically changed the management of axSpA and its associated extra-articular manifestations. Two classes of biologics have demonstrated their efficacy for the treatment of axSpA: the tumor necrosis factor inhibitors [anti-TNF] including four anti-TNF monoclonal antibodies [mAb], infliximab [IFX], adalimumab [ADA], golimumab [GOL], and certolizumab [CTZ], and one soluble receptor fusion, etanercept [ETN], and the interleukin-17A inhibitors [anti-IL17A] including secukinumab [SCK] and ixekizumab [IXE].

The efficacy of biologics on axSpA-associated AAU is less well known. Among anti-TNF mAb, ADA has been shown to significantly reduce the risk of non-infectious uveitic flare, associated or not with axSpA, in two placebo-controlled RCTs [5, 6]. Several observational studies and post-hoc analysis from placebo-controlled studies focused on axSpA-associated AAU and showed that the incidence of AAU decreases after treatment with anti-TNF mAb compared to the incidence before treatment [7–13]. These studies mainly involved IFX and ADA. However, a meta-analysis of 8 RCTs concluded that anti-TNF mAb were not associated with fewer AAU flares, including relapses and new onset, than placebo [14].

The effect of receptor fusion proteins including ETN on AAU associated with axSpA is even more unclear. The before/after treatment rates of AAU in 1365 patients with AS from the Swedish biologics register concluded to a significant increase with ETN [9], also other observational studies did not observe any difference [7, 15]. At the opposite, Wu et al. reported a preventive effect of ETN for flares [relapse or new onset] of uveitis in AS patients in their meta-analysis [14].

Data is scarce on anti-IL17A on axSpA-associated AAU or HLA-B27-associated AAU. Two randomized, double-blinded, placebo-controlled studies assessing the efficacy of SCK in non-infectious non-Behcet’s uveitis showed no difference in the recurrence rate or in reducing intraocular inflammation versus placebo [16]. A pooled data analysis of three RCTs reviewed 794 patients with AS, of whom 135 had uveitis history, and reported an exposure-adjusted incidence rate of 0.03 per 100 patients-years [17]. The same type of pooled analysis of the COAST-V and COAST-W studies was performed on data from 641 patients with AS who received at least one injection of ixekizumab [18]. Across both studies, 20.1% of patients had a history of AAU, and 20 reported AAU flare, 15 of those had a prior history of AAU. The exposure-adjusted rate of AAU was 3.9 per patients-years.

Thus, association between biologic in axSpA and AU manifestations remains poorly defined. Therefore, we aimed to conduct a systematic review and a pairwise and a network meta-analysis to help clinical practice by comparing different biologics on their protecting effect on AU flares.

## Methods

### Data sources and search strategy

A systematic search of the literature was conducted in MEDLINE [via PubMed], EMBase, and the Cochrane Library from inception to May 3, 2020.

We used the following search strategy in PubMed: [“spondylitis, ankylosing” [MeSH Terms] OR spondylart*[tiab] OR spondyloa*[tiab]] AND [“infliximab” [MeSH Terms] OR “certolizumab pegol” [MeSH Terms] OR “etanercept” [MeSH Terms] OR “adalimumab” [MeSH Terms] OR “golimumab” [tw] OR “secukinumab” [tw] OR “ixekizumab” [tw] OR “infliximab” [tw] OR “certolizumab” [tw] OR “etanercept” [tw] OR “adalimumab” [tw]] AND “randomized”] and the following search strategy in EMBase: *[“ankylosing spondylitis”/exp OR “axial spondylarthritis”/exp OR “spondylarthritis”/exp] AND [“etanercept”/exp OR “infliximab”/exp OR “adalimumab”/exp OR “golimumab”/exp OR “certolizumab pegol”/exp OR certolizumab OR “secukinumab”/exp OR “ixekizumab”/exp] AND [“randomized controlled trial”/de].*

Our search concerned articles published in English. A hand search was also performed. Finally, we collected data from electronic abstract databases of the annual scientific meetings of the EUropean League Against Rheumatism Rheumatology congress and the American College of Rheumatology from 2016 to 2019.

### Study selection

Two authors [DR, MB] independently determined the eligibility of the studies after reading title, keywords and abstract. Discrepancy was resolved by consensus.

We pre-specified the target population, interventions, comparators, outcome measures of interest, and timing, following the PICOTs framework.

Inclusion criteria for full text were: 1) RCT published in English before May 3, 2020; 2) comparing the efficacy of any anti-TNF or anti-IL17A versus a comparator (placebo or another active treatment); 3) in a study population of patients with axSpA according to the Assessment of SpondyloArthritis International Society (ASAS) [19] or modified New York (mNY) criteria [20]; 4) with data provided on the number of AU occurring during the controlled period in the safety chapter.

We applied the following exclusion criteria in a sequential order: (1) duplicates (between 2 electronic databases, or in a same electronic database but between 2 different journals), (2) language not English, (3) off topic, (4) design other than RCT, (5) population (not axSpA, or patients under the age of 18, or wrong classification criteria), (6) inadequate comparison, and (7) inadequate safety data reporting (adverse events had to be noticed during the controlled period, separately from the open-label extension).

We considered all five currently available anti-TNF (ADA, ETN, IFX, GOL, CTZ) and two anti-IL17A (SCK, IXE). We included open-labeled controlled studies only if they included an initial double-blind period with detailed safety analysis during this period.

### Data extraction and study quality assessment

Two investigators (DM, MB) independently extracted all data using a standardized spreadsheet and assessed the quality of evidence using the Cochrane risk-of-bias (RiOB) 2.0 tool. Discrepancy was resolved by consensus. For each article, we collected according a pre-specified strategy the following information: age and gender, disease symptoms duration, percentage of history of uveitis, concomitant use of conventional synthetic disease-modifying antirheumatic drugs (csDMARDs), corticosteroids or NSAIDs at baseline, study design, inclusion criteria, dosage and schedule of the treatment, duration of the RCT and/or of the double-blind period, and sample size. For all extracted data, a central value (mean or median) and variability (standard deviation or interquartile range) were collected.

### Outcomes

Our primary outcome was the AU flares incidence, including relapses and new onsets. We collected the number of AU for each clinical trial and each group of treatment and control, by taking into account the safety population. We took into account the terms “uveitis” and “iritis” for AU events. For the studies including a double-blind period and an open-labeled period, only the data from the double-blind period were extracted.

### Statistical analyses

#### Pairwise meta-analysis

All pairwise meta-analyses were performed using the inverse variance approach, which assumes a fixed effect model, to determine the weight given to each study [21], with a direct comparison of the risk of AAU events with each biologic treatment, categorized by anti-TNF mAb, ETN, anti-IL17A and placebo. We also conducted supplementary subgroups analysis: a) According to the axSpA phenotype: RCTs with only patients with AS according to the mNY criteria versus RCTs including both nr-axSpA and AS patients according to the ASAS criteria; b) According to the disease duration: RCTs with only early axSpA, i.e. < 5 years since onset of symptoms, versus RCTs with non-early axSpA; c) According to the trial quality: RCTs with overall low risk of bias using the Cochrane RiOB 2.0 Tool versus RCTs with moderate or high risk of bias and d) According to the focus on AAU: RCTs with mentioned AAU history versus RCTs without detailed information on AAU history.

We measured heterogeneity across studies using the Cochran’s Q test, and I^2^ statistic, with higher values reflecting increasing heterogeneity. We assessed publication bias by examining funnel plots and using the Egger’s regression asymmetry test.

The statistical analysis was performed with Comprehensive Meta-Analysis Version 3 [22].

#### Network meta-analysis

We also conducted a network meta-analysis to perform an adjusted indirect comparison of the investigational treatment arms categorized in 3 different groups of treatment (anti-TNF mAb, ETN and anti-IL17A). The results of these meta-analyses are the odds ratio (OR) of AU events between treatments (i.e., anti-TNF mAb, ETN and anti-IL17A and placebo) with their 95% confidence interval and the statistical significance level of the comparison. The homogeneity and consistency assumption was based on a generalized Cochran’s Q statistic for multivariate meta-analysis. We ranked the efficacy of the 3 different groups of treatment and placebo, using P-scores that measure the mean extent of certainty that a treatment is better than the competing treatment. In our study, the higher the P-score, the more effective the treatment was in preventing AU flares. The assessment of publication bias was made using funnel plots in this multiple treatment comparison.

The statistical analysis was performed using R statistical packages (version 3.2.4) and the meta-library, Netmeta [20].

## Results

### Eligible studies

**Fig. 1:**
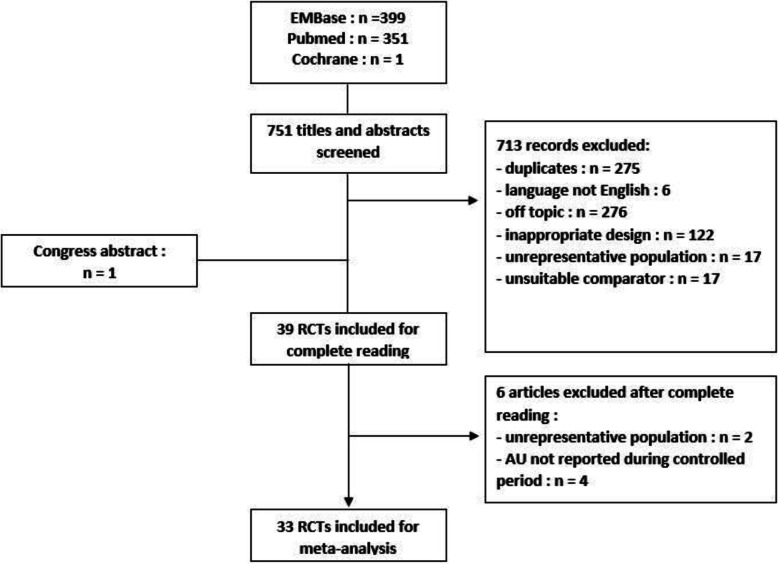
Study selection process (flowchart). RCT, randomized controlled trial; AU, anterior uveitis

**Fig. 2:**
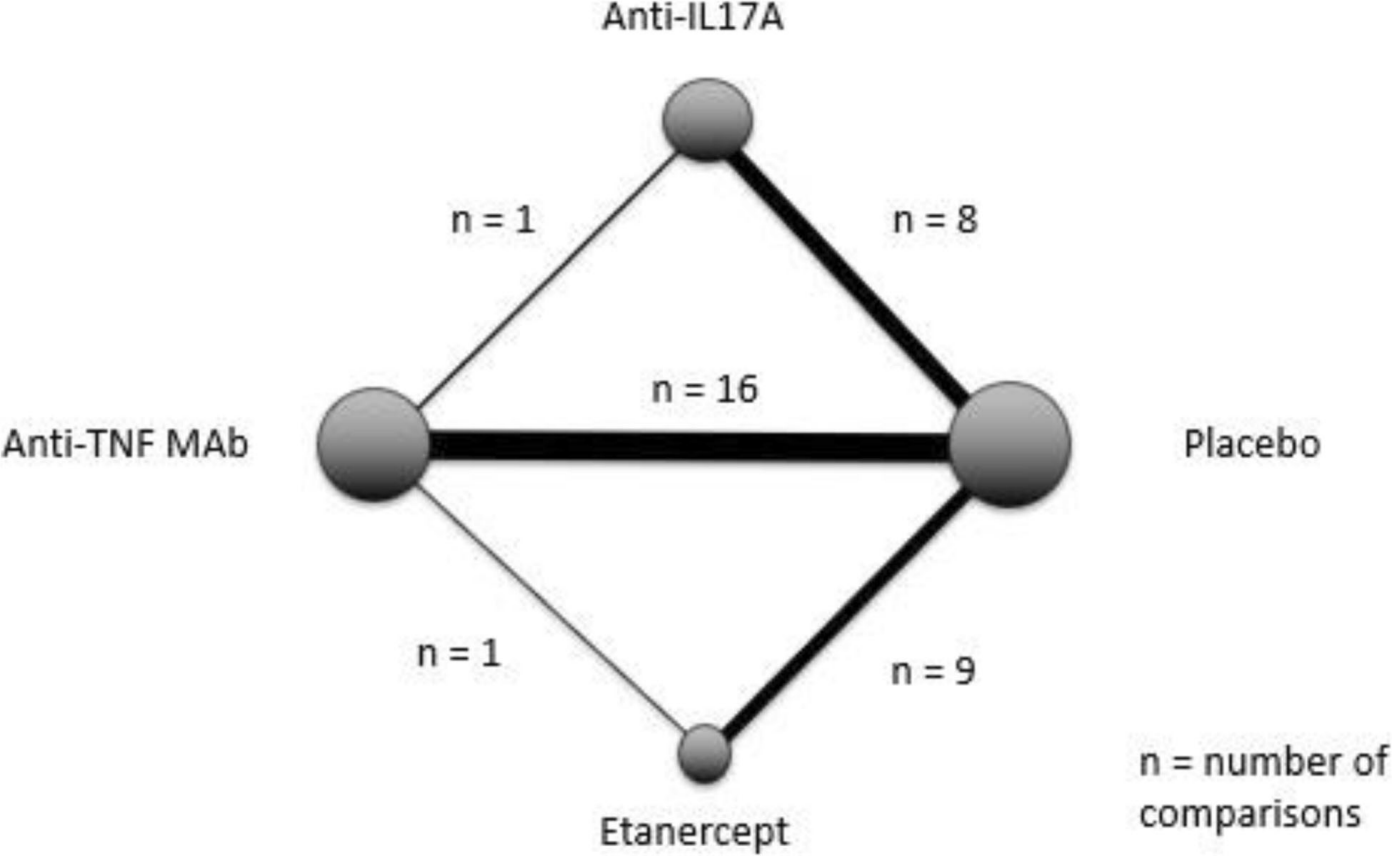
Network configuration of eligible comparisons. Nodes represent each intervention with a size proportional to the number of patients receiving treatment. Lines represent direct comparisons; the more patients involved in comparison, the thicker the line. TNF, tumor necrosis factor; mAb, monoclonal antibody; ETN, etanercept; IL17A, interleukin-17A

We identified 751 citations after search in the 3 databases plus one more after search in the congress abstract databases (Fig. 1). After reading titles and abstracts, we excluded 713 abstracts, mainly because of duplicates or off topic studies. After the complete reading, we excluded 6 articles: 4 because AU were not described during the controlled period and 2 for an unrepresentative population. Finally, we included 33 RCTs (Figs. 1 and 2), allowing 35 comparisons, comprising 4544 patients with axSpA treated with a biologic treatment and 2497 treated with placebo.

### Study characteristics

The Additional file 1 provides detailed characteristics of the 33 RCTs included in the analyses. A comparison of a biologic treatment versus placebo was performed in 32 placebo-controlled RCTs [23–55], including one RCT comparing 2 biologic treatments, i.e. IXE and ADA, versus placebo [26], and in a head-to-head RCT comparing IFX versus ETN [39]. Anti-TNF mAb were assessed in 17 RCTs (ADA: 4 [23–26]; CTZ: 2 [27, 28]; GOL: 4 [29–32]; IFX: 7 [33–39]), ETN was assessed in 10 RCTs [39–48] and anti-IL17A were assessed in 8 RCTs (SCZ: 5 [49–53]; IXE: 3 [26, 54, 55]. The mean duration of the controlled period was 22.7 weeks ±18.5 (SD), median: 16 weeks (range: 6–104 weeks).

According to the Cochrane RiOB 2.0 tool, 16 RCTs had a low risk of bias, 17 RCTs presented some concerns, and none had a high risk of bias (Additional file 2).

### Characteristics of the patients with axSpA

The main characteristics of the intention-to-treat (ITT) population are summarized in Table 1. Because our objective was to collect the AU flares, we included in the analysis the safety populations. A total of 7041 patients were included in the analysis, of whom 2497 received a placebo, 2101 were treated with an anti-TNF mAb, 699 with ETN, and 1744 with an anti-IL17A, with a follow-up of 3264 patient years. The total cumulative exposure under active treatment was 2265 patient years.

**Table 1:** AAU Flare reported in the included RCTs

Study (ref)			Treatment			Placebo		
	Treatment	Study duration (weeks)	Patients (n)	Uveitis (n)	Incidence (per 100 PY)	Patients (n)	Uveitis (n)	Incidence (per 100 PY)
Van der Heijde D et al. 2006 [23]	ADA	24	208	0	0.00	107	0	0.00
Sieper J et al. 2012 [24]	ADA	12	95	0	0.00	97	0	0.00
Huang F et al. 2013 [25]	ADA	12	229	0	0.00	115	0	0.00
Landewé R et al. 2013 [27]	CTZ	24	218	2	1.98	107	3	6.07
Deodhar A et al. 2019 [28]	CTZ	52	159	4	2.51	158	8	5.06
Inman R et al. 2008 [29]	GOL	24	278	0	0.00	77	0	0.00
Deodhar A et al. 2017 [32]	GOL	16	105	0	0.00	103	2	6.31
Sieper J et al. 2015 [31]	GOL	16	97	0	0.00	100	0	0.00
Bao C et al. 2014 [30]	GOL	24	169	1	1.28	44	0	0.00
Van der Heijde D et al. 2005 [34]	IFX	18	202	0	0.00	75	0	0.00
Barkham N et al. 2009 [36]	IFX	16	20	0	0.00	20	0	0.00
Inman R et al. 2010 [37]	IFX	12	39	0	0.00	37	0	0.00
Marzo-Ortega H et al. 2005 [35]	IFX	30	28	1	6.19	14	0	0.00
Sieper J et al. 2012 [38]	IFX	28	105	0	0.00	52	0	0.00
Braun J et al. 2002 [33]	IFX	12	34	1	12.74	35	3	37.14
Gorman JD et al. 2002 [40]	ETN	16	20	0	0.00	20	0	0.00
Davis JC et al. 2003 [41]	ETN	24	138	3	4.71	139	8	12.47
Brandt J et al. 2003 [42]	ETN	6	14	0	0.00	16	0	0.00
Calin A et al. 2004 [43]	ETN	12	45	0	0.00	39	0	0.00
Van der Heijde D et al. 2006 [44]	ETN	12	305	0	0.00	51	1	8.49
Barkham N et al. 2010 [45]	ETN	12	15	0	0.00	17	0	0.00
Dougados M et al. 2011 [46]	ETN	12	39	0	0.00	43	0	0.00
Dougados M et al. 2014 [48]	ETN	8	42	0	0.00	48	0	0.00
Dougados M et al. 2014 [47]	ETN	12	106	0	0.00	109	0	0.00
Deodhar A et al. 2019 [54]	IXE	16	212	5	7.66	104	0	0.00
Van der Heijde D et al. 2018 [26]	IXE	16	164	1	1.98	86	0	0.00
Deodhar A et al. 2019 [55]	IXE	52	198	3	2.09	104	2	1.92
Baeten D et al. 2013 [49]	SEC	28	24	0	0.00	6	0	0.00
Baeten D et al. 2015 [50]	SEC	16	394	7	5.77	196	2	3.31
Pavelka K et al. 2017 [51]	SEC	16	150	0	0.00	75	0	0.00
Kivitz A et al. 2018 [52]	SEC	16	233	0	0.00	117	0	0.00
Deodhar A et al. 2019 [53]	SEC	52	369	7	1.21	186	2	1.07
Giardina A et al. 2010 [39]	IFX	104	25	1	2.00	-	-	-
	ETN		25	2	4.00	-	-	-

The different treatment groups were similar in terms of gender, age, duration of symptoms, Bath Ankylosing Spondylitis Disease Activity Index (BASDAI) at baseline, concomitant csDMARD, non-steroidal anti-inflammatory drugs (NSAIDs), and corticosteroids intake. AU and inflammatory bowel disease (IBD) history were less frequent in the ETN and anti-IL17A groups than in the anti-TNF mAb.

### Annual Incidence of AU

A total of 69 AU flares (de novo or relapses) was reported during the controlled periods: 31 among placebo-treated patients, 10 among anti-TNF mAb-treated patients (CTZ 6, IFX 3, and GOL 1), 5 among ETN-treated patients, and 23 among anti-IL17A-treated patients (SCK 14 and IXE 9) (Table 1). Crude annual incidence of AU was 1.06%, 2.14%, 2.11%, and 3.10%, in the anti-TNF mAb, ETN, anti-IL17A, and placebo groups, respectively.

### Pairwise meta-analysis

**Fig. 3:**
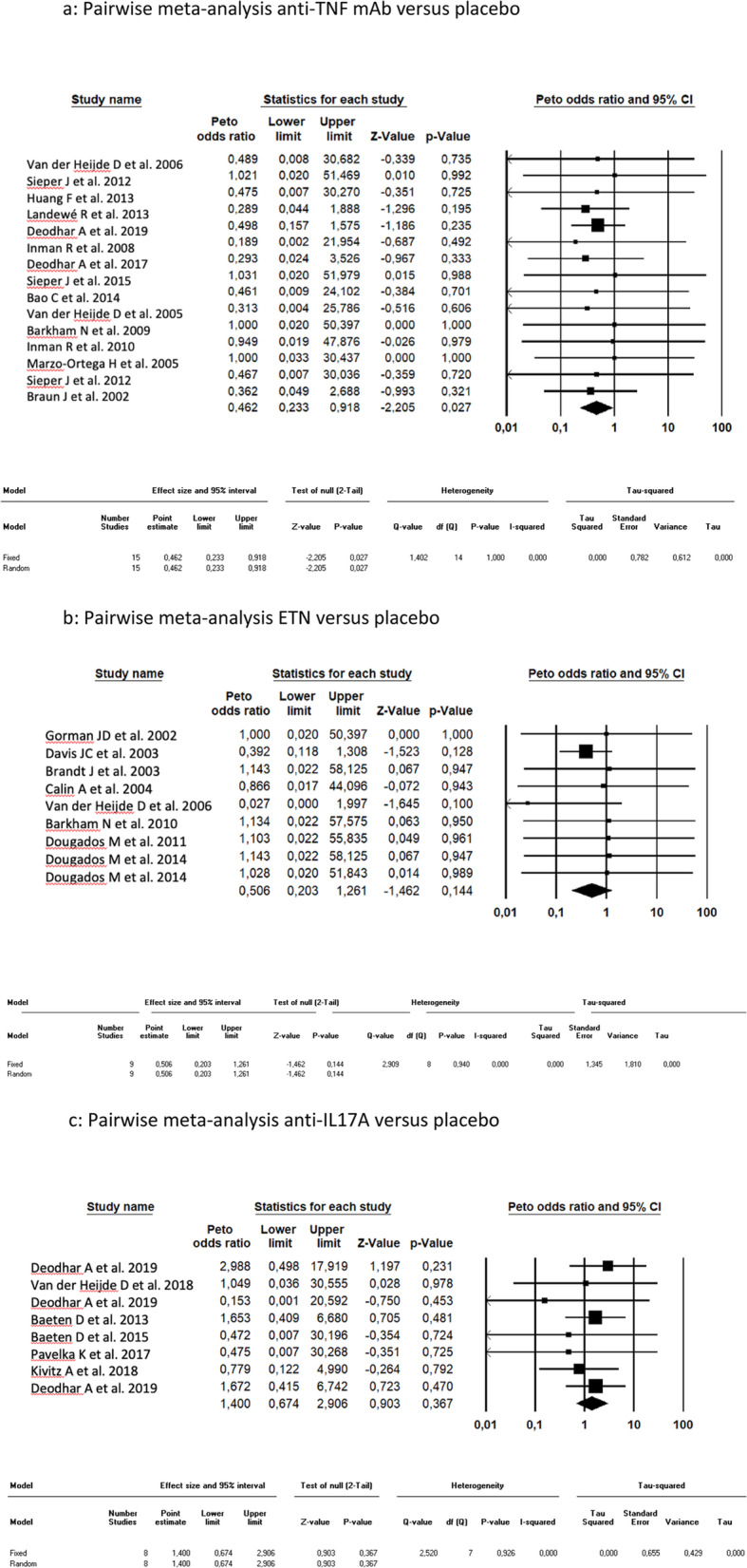
Pairwise meta-analysis. **a** Pairwise meta-analysis anti-TNF mAb versus placebo. **b** Pairwise meta-analysis ETN versus placebo. **c** Pairwise meta-analysis anti-IL17A versus placebo. TNF, tumor necrosis factor; mAb, monoclonal antibody; ETN, etanercept; IL17A, interleukin-17A; CI, confidence interval

The AU incidence reported in patients with axSpA treated with anti-TNF mAb was significantly lower than with placebo (OR = 0.499, CI 95% [0.256–0.973] *p* = 0.041). There was no significant difference in AU incidence between ETN (OR = 0.499, CI 95% [0.198–1,259] *p* = 0.141) or anti-IL17A (OR = 1,345, CI 95% [0.465–3,886] *p* = 0.585) and placebo (Fig. 3a–c). No publication bias is suggested according to the Egger’s regression test (*p* = 0.308) for each category of biologic treatment.

Subgroup analyses following pre-specified criteria to compare the incidence of uveitis in each subgroup for each biologic versus placebo showed no significant differences according to axSpA phenotype, disease duration, risk of bias, or focus on AU history.

### Network meta-analysis

**Fig. 4:**
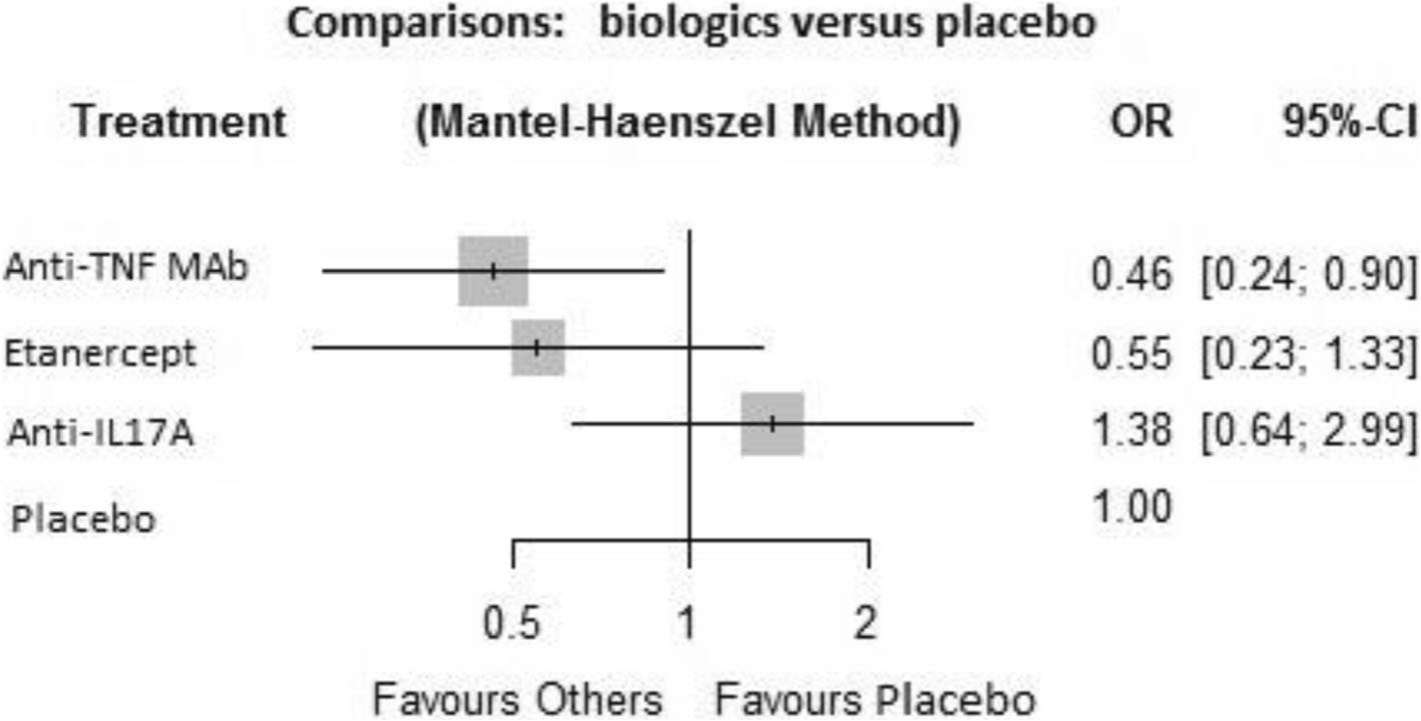
Forest plots of network meta-analysis of all trials for AAU incidence. AAU, acute anterior uveitis; TNF, tumor necrosis factor; mAb, monoclonal antibody; IL17A, interleukin-17A; OR, odds ratio; CI, confidence interval

Incidence of AU flares was lower with anti-TNF mAb compared to placebo (OR = 0.46; IC 95% [0.24–0.90]) (Fig. 4). There was also a significant difference for a decreased incidence of AU with anti-TNF mAb compared to anti-IL17A (OR = 0.34; CI 95% [0.12–0.92]) (Table 2). The other comparisons between biologics or between biologics and placebo were not significant (Table 2). The Cochran’s Q test was 0.57 (*p* = 0.903) ascertaining the absence of heterogeneity/inconsistency between RCTs included.

**Table 2:**
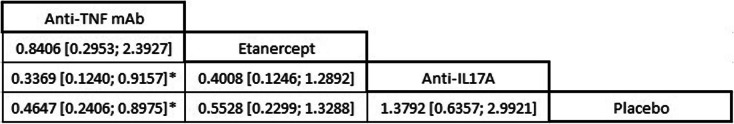
Comparison for the preventive effect on AAU flares (OR and 95% CI)

*OR* Odd-Ratio, *CI* Confidence Interval, *AU* Anterior Uveitis, *TNF* Tumor Necrosis Factor, *mAb* monoclonal antibody, *IL17A* interleukin-17A

^*^p < 0.05

P-scores that measure the mean extent of certainty that a treatment is better than the competing treatments were 0.86, 0.728, 0.274, and 0.137 in the anti-TNF mAb, ETN, placebo, and anti-IL17A groups, respectively. Ranking treatments by using P-scores suggested that incidence of AU was the lowest with anti-TNF mAb and the highest with anti-IL17A.

The examination of the funnel plot does not provide suspicion of an asymmetrical distribution of the points representing the studies.

## Discussion

This study of 33 RCTs is, to our knowledge, the first network meta-analysis comparing incidence of AU in both anti-TNF, anti-IL17A, and placebo. Flares of AU were uncommon whatever the treatment with a total of 38 AU events reported under active treatment during controlled periods, for a total cumulative exposure under active treatment of 2265 patient years. Despite this low incidence, our results showed a significant protective effect on AU flares of anti-TNF mAb compared to placebo and compared to anti-IL17A.

The reduction of AAU incidence rate with anti-TNF mAb compared to placebo or before/after treatment has already been described in various observational studies for IFX, ADA and GOL [7–10, 12, 13]. However, unlike in our study, a previous pairwise meta-analysis did not report a protective effect of anti-TNF mAb on AAU flares versus placebo [OR: 0.43, 95% CI: 0.12–1.49, *p* = 0.18] [14]. This discrepancy can be explained by differences in the inclusion criteria. The Wu et al. pairwise meta-analysis selected RCTs including only patients with AS and RCTs with a follow-up > 12 weeks. When applying our inclusion criteria until February 2014 (limit of their meta-analysis research), we would have included 18 RCTs instead of the 8 RCTs included in their analysis.

The same pairwise meta-analysis [14] suggested a protective effect on AAU flares of ETN compared to placebo, while we did not find any significant difference between the ETN and placebo groups. Our results are confirmed by the conclusions of two randomized placebo-controlled clinical trials where etanercept did not show superiority to placebo in preventing flare rates of uveitis [56, 57]. As well, the analysis of a large US claims database aimed at comparing the risk of developing uveitis in patients initiating anti-TNF in patients with AS [58] showed that adalimumab and infliximab were associated with a lower incidence of uveitis episodes than etanercept: 2.4% for adalimumab, 3.2% for infliximab and 4.5% for etanercept.

While our results showed that anti-IL17A do not have the same protective effect against AU flares as anti-TNF mAb, they are also reassuring with regard to a possible deleterious effect of anti-IL17 without significant difference between anti-IL17 and placebo (OR = 1.38 [CI 95% 0.63–2.99]). Among anti-IL17A, the incidence rate was 1.69 per 100 PY with SCK and 3.47 per 100 PY with IXE. Incidence of AU with anti-IL17A in axSpA has recently been published in a pooled analysis of 3 RCTs [53] assessing SCK with an incidence rate of 1.4 per 100 PY, close to our results.

In the pairwise meta-analysis, subgroup analyses did not show any interaction of axSpA phenotype, disease duration, or AU history reporting on AU incidence under anti-TNF MAb, ETN, or anti-IL17A. In other words, we did not find difference between AS and nr-axSpA, between recent and non-recent ax-SpA and between patients with or without AU history.

The methodological quality of this meta-analysis relies on a double lecture aiming at limiting the risk of errors in selecting studies and extracting data. We obtained 33 homogeneous RCTs, with no publication bias according to funnel plots. This broad selection of RCTs has provided us a total cumulative exposure of 3264 PY. However, despite the number of RCTs included, few comparisons between two active treatments or between an active treatment and placebo are not significant. The absence of statistical difference between different biologic therapies may reflect a real lack of clinical effect but can also be due to a lack of power. This lack of power results from the choice to perform a network meta-analysis imposed to limit extraction of data to the controlled periods. The median duration of placebo (or active)-controlled period was 16 weeks, which limits the risk of rare events such as AU.

## Conclusion

In RCTs assessing treatments in axSpA, incident AU are rare events.

This network meta-analysis demonstrates that anti-TNF mAb are associated with a lower incidence of AU flare compared to placebo and to anti-IL17A. The incidence of AU was not increased with anti-IL17A or ETN compared to placebo.

## Supplementary Information

**Additional file 1.** Characteristics of randomized controlled trials included in this meta-analysis.

**Additional file 2.** Risk of bias in included RCTs (Cochrane risk of bias 2.0 tool).

**Additional file 3.** Funnel plots.

**Additional file 4.** Subgroup analysis.

**Additional file 5.** Funnel plots.

## Data Availability

The datasets analyzed during the current study are available from the corresponding author on reasonable request.
