# Supplementary material for: Incidence of anterior uveitis in patients with axial spondyloarthritis treated with anti-TNF or anti-IL17A: a systematic review, a pairwise and network meta-analysis of randomized controlled trials
Source: Arthritis Res Ther. 2021 Jul 16;23:192. doi: 10.1186/s13075-021-02549-0 (PMC8283999; doi:10.1186/s13075-021-02549-0)
Supplement: Supplementary file 1 — Additional file 1. Characteristics of randomized controlled trials included in this meta-analysis. [file 13075_2021_2549_MOESM1_ESM.docx]

Additional file 1: Characteristics of randomized controlled trials included in this meta-analysis.

| **First author** | **Year** | **Treatment** | **n** | **Control** | **n** | **Study protocol with doses** | **Duration (wk)** |
| --- | --- | --- | --- | --- | --- | --- | --- |
| Van der Heijde D. | 2006 | Adalimumab | 208 | Placebo | 107 | ADA 40 mg / wk Placebo | 24 |
| Sieper J. | 2012 | Adalimumab | 95 | Placebo | 97 | ADA 40 mg / wk Placebo | 12 |
| Huang F. | 2013 | Adalimumab | 229 | Placebo | 115 | ADA 40 mg / wk Placebo | 12 |
| Landewé R. | 2013 | Certolizumab | 218 | Placebo | 107 | CZP 200 mg / 2 wk CZP 400 mg / 4 wk  Placebo | 24 |
| Deodhar A. | 2019 | Certolizumab | 159 | Placebo | 158 | CZP 400 mg at wk 0, 2 and 4 then CZP 200 mg / 2 wk Placebo | 52 |
| Inman A. | 2008 | Golimumab | 278 | Placebo | 77 | GOL 50 mg / 4 wk GOL 100 mg / 4 wk  Placebo | 24 |
| Deodhar A. | 2017 | Golimumab | 105 | Placebo | 103 | GOL IV 2 mg/kg at wk 0, 4, 12 and then / 8 wk Placebo | 28 |
| Sieper J. | 2015 | Golimumab | 97 | Placebo | 100 | GOL 50 mg / 4 wk Placebo | 16 |
| Bao C. | 2014 | Golimumab | 169 | Placebo | 44 | GOL 50 mg / 4 wk  Placebo | 16 |
| Van der Heijde D. | 2005 | Infliximab | 202 | Placebo | 75 | IFX 5 mg/kg at wk 0, 2, 6, 12, 18 Placebo | 24 |
| Barckham N. | 2009 | Infliximab | 20 | Placebo | 20 | IFX 5 mg / kg at wk 0, 2, 6, 12 Placebo | 12 |
| Inman R. | 2010 | Infliximab | 39 | Placebo | 37 | IFX 3 mg / kg at wk 0, 2, 6 Placebo | 12 |
| Marzo-Ortega H. | 2005 | Infliximab | 28 | Placebo | 14 | IFX 5 mg / kg at wk 0, 2, 6, 14 and 22 Placebo | 30 |
| Sieper J. | 2012 | Infliximab | 105 | Placebo | 52 | IFX 5 mg / kg at wk 0, 2, 6, 12, 18 and 24 Placebo | 28 |
| Braun J. | 2002 | Infliximab | 34 | Placebo | 35 | IFX 5 mg / kg at wk 0, 2, 6 | 12 |
| Gorman J. | 2002 | Etanercept | 20 | Placebo | 20 | ETN 25mg 2x/wk Placebo | 16 |
| Davis JC. | 2003 | Etanercept | 138 | Placebo | 139 | ETN 25mg 2x/wk Placebo | 24 |
| Brandt J. | 2003 | Etanercept | 14 | Placebo | 16 | ETN 25mg 2x/wk Placebo | 12 |
| Calin A. | 2004 | Etanercept | 45 | Placebo | 39 | ETN 25mg 2x/wk Placebo | 12 |
| Van der Heijde D. | 2006 | Etanercept | 305 | Placebo | 51 | ETN 50mg/wk ETN 25mg/wk Placebo | 12 |
| Barckham N. | 2010 | Etanercept | 15 | Placebo | 17 | ETN 25mg 2x/wk Placebo | 12 |
| Dougados M. | 2011 | Etanercept | 39 | Placebo | 43 | ETN 50mg/wk Placebo | 12 |
| Dougados M. | 2014 | Etanercept | 42 | Placebo | 48 | ETN 50mg/wk Placebo | 8 |
| Dougados M. | 2014 | Etanercept | 106 | Placebo | 109 | ETN 50mg/wk Placebo | 12 |
| Deodhar A. | 2019 | Ixekizumab | 212 | Placebo | 104 | IXEQ 80mg/2wk IXEQ 80mg/4wk Placebo | 16 |
| Van der Heijde D. | 2018 | Ixekizumab/ Adalimumab | 164 IXE 90 ADA | Placebo | 86 | IXEQ 80mg/2wk IXEQ 80mg/4wk ADA 40mg/2wk Placebo | 16 |
| Deodhar A. | 2019 | Ixekizumab | 198 | Placebo | 104 | IXE 80mg/2wk  IXE 80mg/4wk  Placebo | 52 |
| Deodhar A. | 2019 | Secukinumab | 369 | Placebo | 186 | SCK 150mg at wk 0,1,2,3 and then /4wk  SCK 150mg at wk 0 and then /4wk  Placebo | 52 |
| Baeten D. | 2013 | Secukinumab | 24 | Placebo | 6 | SCK IV 10 mg/kg /3wk Placebo | 28 |
| Baeten D. | 2015 | Secukinumab | 394 | Placebo | 196 | SCK IV 10 mg/kg /2wk 3x then  150 mg/4wk SCK IV 10 mg/kg /2wk 3x then 75 mg/4wk SCK 150 mg at wk 0,1,2,3 and then /4wk SCK 75 mg at wk 0,1,2,3 and then /4wk Placebo | 16 |
| Pavelka K. | 2017 | Secukinumab | 150 | Placebo | 75 | SCK IV 10 mg/kg /2wk 3x then  150 mg/4wk SCK IV 10 mg/kg /2wk 3x then 75 mg/4wk Placebo | 16 |
| Kivitz A. | 2018 | Secukinumab | 233 | Placebo | 117 | SCK 150 mg at wk 0,1,2,3 and then /4wk with loading dose SCK 150 mg at wk 0,1,2,3 and then /4wk Placebo | 16 |
| Giardina A. | 2010 | Infliximab | 25 | Etanercept | 25 | IFX 5 mg/wk at wk 0,2,6 and /6wk ETN 50mg/wk | 12 |

Wk : weeks
