# Supplementary figures and images for: Incidence of anterior uveitis in patients with axial spondyloarthritis treated with anti-TNF or anti-IL17A: a systematic review, a pairwise and network meta-analysis of randomized controlled trials
Source: Arthritis Res Ther. 2021 Jul 16;23:192. doi: 10.1186/s13075-021-02549-0 (PMC8283999; doi:10.1186/s13075-021-02549-0)

Additional File 2: Risk of bias in included RCTs (Cochrane risk of bias 2.0 tool)


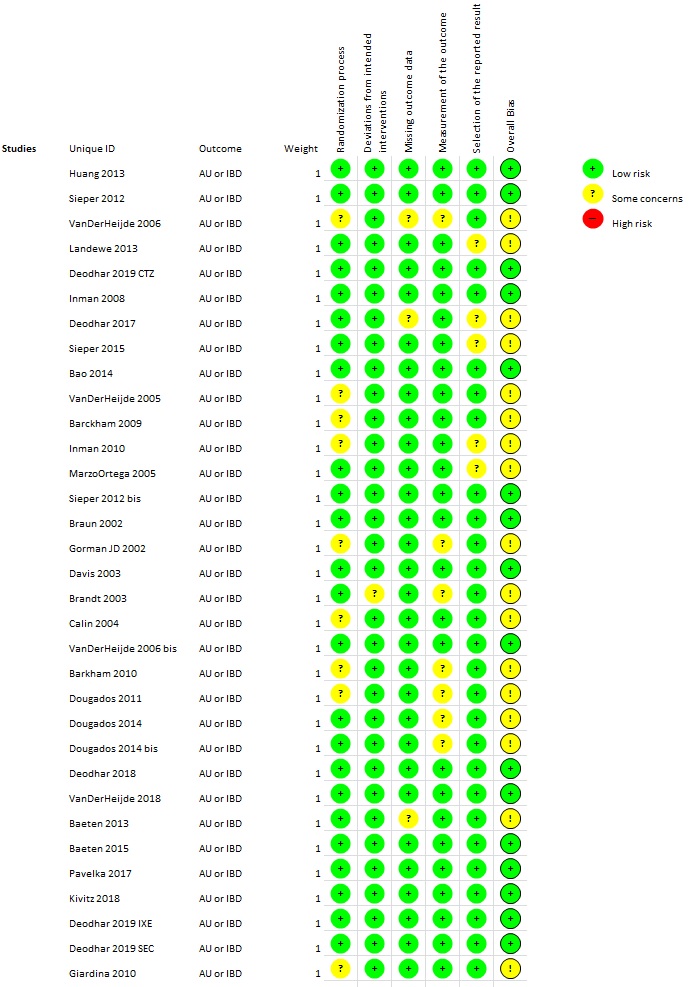

Supplement: Supplementary file 2 — Additional file 2. Risk of bias in included RCTs (Cochrane risk of bias 2.0 tool). [file 13075_2021_2549_MOESM2_ESM.docx]

Additional File 5: Funnel plots


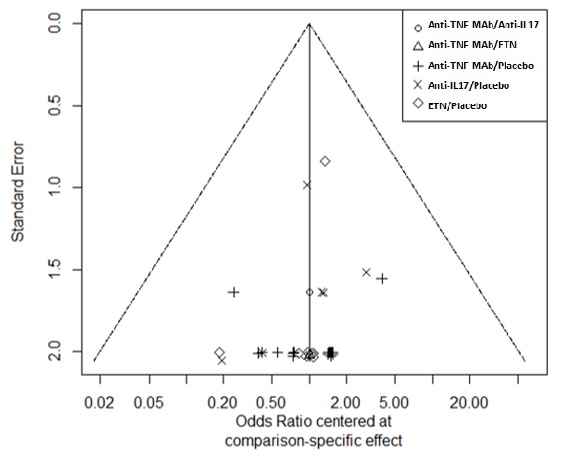

Supplement: Supplementary file 5 — Additional file 5. Funnel plots. [file 13075_2021_2549_MOESM5_ESM.docx]
