# Supplementary material for: Incidence of anterior uveitis in patients with axial spondyloarthritis treated with anti-TNF or anti-IL17A: a systematic review, a pairwise and network meta-analysis of randomized controlled trials
Source: Arthritis Res Ther. 2021 Jul 16;23:192. doi: 10.1186/s13075-021-02549-0 (PMC8283999; doi:10.1186/s13075-021-02549-0)
Supplement: Supplementary file 3 — Additional file 3. Funnel plots. [file 13075_2021_2549_MOESM3_ESM.docx]

Additional File 3: Baseline characteristics of patients with axSpA (ITT population)

| Study | Trt | Ctrl | N total | N Trt | N Ctrl | Gender ; (Male, n) | | Age, (year, mean) | | HLA B27 (positive, n | | History of MICI (n) | | History of Uveitis (n) | | Symptoms duration (year, mean) | | BASDAI, mean | | csDMARDs (n) | | Corticosteroids (n) | |
| --- | --- | --- | --- | --- | --- | --- | --- | --- | --- | --- | --- | --- | --- | --- | --- | --- | --- | --- | --- | --- | --- | --- | --- |
|  |  |  |  |  |  | Trt | Ctrl | Trt | Ctrl | Trt | Ctrl | Trt | Ctrl | Trt | Ctrl | Trt | Ctrl | 6,3 | 6,3 | 40 | 26 | 25 | 6 |
| Van der Heijde D et al. 2006 | ADA | PCB | 315 | 208 | 107 | 157 | 79 | 41,7 | 43,4 | 163 | 85 | 15 | 2 | 68 | 27 | 11,3 | 10 | 6,4 | 6,5 | 17 | 16 | - | - |
| Sieper J et al. 2012 | ADA | PCB | 185 | 91 | 94 | 44 | 40 | 37,6 | 38,4 | 75 | 70 | 4 | 6 | 12 | 10 | 10,1 | 10,1 | 6 | 6,2 | 134 | 70 | 8 | 5 |
| Huang F et al. 2013 | ADA | PCB | 344 | 229 | 115 | 185 | 95 | 30,1 | 29,6 | 219 | 109 | 1 | 0 | 5 | 5 | 8,1 | 7,7 | 6,5 | 6,5 | 62 | 38 | - | - |
| Landewé R et al. 2013 | CTZ | PCB | 325 | 218 | 107 | 135 | 65 | 39,4 | 39,9 | 168 | 87 | - | - | - | - | 7,4 | 7,7 | 6,9 | 6,8 | 55 | 48 | 16 | 16 |
| Deodhar A et al. 2019 | CTZ | PCB | 317 | 159 | 158 | 78 | 76 | 37,3 | 37,4 | 128 | 132 | - | - | 28 | 36 | 7,8 | 8 | 6,8 | 6,6 | 130 | 41 | 44 | 13 |
| Inman A et al. 2008 | GOL | PCB | 356 | 278 | 78 | 200 | 55 | 38 | 41 | 230 | 66 | 18 | 8 | 58 | 25 | 11 | 16 | 7 | 7,1 | 56 | 60 | 32 | 23 |
| Deodhar A et al. 2017 | GOL | PCB | 208 | 105 | 103 | 86 | 77 | 38,4 | 39,2 | - | - | - | - | - | - | 10,2 | 11,6 | 6,6 | 6,4 | - | - | - | - |
| Sieper J et al. 2015 | GOL | PCB | 198 | 98 | 100 | 61 | 52 | 30,7 | 31,7 | 81 | 82 | - | - | - | - | - | - | 6,6 | 6,5 | 79 | 79 | 5 | 5 |
| Bao C et al. 2014 | GOL | PCB | 213 | 108 | 105 | 90 | 87 | 30,5 | 30,6 | - | - | - | - | - | - | 6,8 | 7,5 | 6,6 | 6,5 | 0 | 0 | 0 | 0 |
| Van der Heijde D et al. 2005 | IFX | PCB | 279 | 201 | 78 | 157 | 68 | 40 | 41 | 173 | 69 | 13 | 6 | 72 | 25 | 7,7 | 13,2 | 5,8 | 5,8 | - | - | - | - |
| Barkham N et al. 2009 | IFX | PCB | 40 | 20 | 20 | 15 | 15 | 29,5 | 28,2 | - | - | - | - | - | - | 1,4 | 1,1 | - | - | - | - | - | - |
| Inman R et al. 2010 | IFX | PCB | 76 | 39 | 37 | 32 | 29 | 42,9 | 39,3 | 28 | 27 | 3 | 4 | 13 | 13 | 18,7 | 18,6 | 6,4 | 6,9 | 28 | 14 | 5 | 3 |
| Marzo-Ortega H. 2005 | IFX | PCB | 42 | 28 | 14 | 23 | 11 | 39 | 41 | 27 | 12 | - | - | - | - | 10 | 8 | - | - | - | - | - | - |
| Sieper J et al. 2012 | IFX | PCB | 156 | 105 | 51 | 72 | 40 | 31,7 | 30,7 | 87 | 47 | 0 | 0 | 6 | 6 | 1,8 | 1,9 | 6,5 | 6,3 | 0 | 0 | 0 | 0 |
| Braun J et al. 2002 | IFX | PCB | 69 | 34 | 35 | 23 | 22 | 40,6 | 39 | 31 | 27 | - | - | 17 | 15 | 16,4 | 14,9 | - | - | 8 | 7 | 5 | 2 |
| Gorman J et al. 2002 | ETN | PCB | 40 | 20 | 20 | 13 | 18 | 38 | 39 | 19 | 18 | - | - | - | - | 15 | 12 | 5,81 | 5,96 | 44 | 43 | 18 | 20 |
| Davis JC et al. 2003 | ETN | PCB | 277 | 138 | 139 | 105 | 105 | 42,1 | 41,9 | 108 | 109 | 7 | 6 | 39 | 43 | 10,1 | 10,5 | 6,5 | 6,6 | 0 | 0 | 0 | 0 |
| Brandt J et al. 2003 | ETN | PCB | 30 | 14 | 16 | 10 | 12 | 39,8 | 32 | 12 | 15 | - | - | 5 | 3 | 14,9 | 11,4 | 6,1 | 5,86 | 16 | 16 | 7 | 6 |
| Calin A et al. 2004 | ETN | PCB | 84 | 45 | 39 | 36 | 30 | 45,3 | 40,7 | - | - | - | - | - | - | 15 | 9,7 | 6,1 | 6,1 | 120 | 17 | 35 | 9 |
| Van der Heijde D et al. 2006 | ETN | PCB | 356 | 305 | 51 | 222 | 40 | 40,7 | 40,1 | - | - | 5 | 4 | 33 | 4 | 9,5 | 8,5 | 6,05 | 5,46 | - | - | 0 | 0 |
| Barkham N et al. 2010 | ETN | PCB | 40 | 20 | 20 | 15 | 17 | 40,8 | 39,4 | - | - | - | - | - | - | 11 | 20 | 6,4 | 5,8 | - | - | - | - |
| Dougados M et al. 2011 | ETN | PCB | 82 | 39 | 43 | 37 | 39 | 46 | 48 | 31 | 36 | - | - | 13 | 12 | 19 | 23 | 6 | 5,9 | - | - | - | - |
| Dougados M et al. 2014 | ETN | PCB | 90 | 42 | 48 | 24 | 32 | 38,8 | 38,9 | 28 | 31 | - | - | 5 | 3 | 6 | 5,5 | 6 | 6 | 21 | 21 | - | - |
| Dougados M et al. 2014 | ETN | PCB | 215 | 106 | 109 | 68 | 62 | 31,9 | 32 | 71 | 83 | 1 | 1 | 8 | 9 | 2,4 | 2,5 | 7,5 | 7,3 | 54 | 33 | 22 | 14 |
| Deodhar A et al. 2019 | IXE | PCB | 316 | 212 | 104 | 166 | 87 | 45,8 | 46,6 | - | - | - | - | - | - | 17,7 | 19,9 | 6,8 | 6,8 | 62 | 31 | - | - |
| Van der Heijde D et al. 2018 | IXE | PCB | 341 | 164 | 87 | 132 | 71 | 41,2 | 42,7 | 150 | 76 | - | - | - | - | 15,8 | 16,6 | 7,15 | 7,2 | 82 | 38 | 28 | 14 |
| Deodhar A et al. 2019 | IXE | PCB | 303 | 198 | 105 | 99 | 44 | 40,4 | 39,9 | 144 | 77 | 1 | 3 | 22 | 12 | 10,9 | 10,1 | 7,01 | 6,76 | 85 | 52 | 31 | 17 |
| Deodhar A et al. 2019 | SEC | PCB | 555 | 369 | 186 | 164 | 91 | 39,5 | 39,3 | 253 | 129 | 5 | 5 | 47 | 18 | 8,65 | 8,39 | 7,1 | 7,2 | 8 | 3 | 3 | 0 |
| Baeten D et al. 2013 | SEC | PCB | 30 | 24 | 6 | 14 | 5 | 41,1 | 45,0 | 16 | 5 | 3 | 1 | 7 | 2 | 10,1 | 10,2 | 6,4 | 6,6 | 160 | 76 | 45 | 23 |
| Baeten D et al. 2015 | SEC | PCB | 590 | 394 | 196 | 269 | 141 | 41,9 | 43,3 | 295 | 148 | 11 | 4 | 61 | 35 | 6,8 | 7,6 | 7 | 6,9 | 57 | 25 | 15 | 14 |
| Pavelka K et al. 2017 | SEC | PCB | 226 | 150 | 76 | 96 | 40 | 42,5 | 42,7 | 108 | 53 | - | - | - | - | 5,6 | 5,2 | 7 | 7,1 | 54 | 37 | 21 | 13 |
| Kivitz A et al. 2018 | SEC | PCB | 350 | 233 | 117 | 164 | 76 | 42,8 | 43,4 | 199 | 93 | 6 | 0 | 44 | 27 | 7,4 | 7,1 | 6,5 | 6,6 | - | - | - | - |
| Giardina A et al. 2010 | IFX | ETN | 50 | 25 | 25 | 19 | 20 | 31,9 | 32,6 | 23 | 24 | - | - | - | - | 15,4 | 15,7 | 6,3 | 6,3 | 40 | 26 | 25 | 6 |

Trt: treatment ; Ctlr: control ; N: number; BASDAI: Bath Ankylosing Spondylitis Disease Activity; csDMARDs: conventional synthetic disease modifying anti rheumatism drug; ADA: adalimumab; PCB: placebo; CTZ: certolizumab; GOL: golimumab; IFX: infliximab ; ETN : etanercept ; SEC : secukinumab ; IXE : ixekizumab
