# Supplementary material for: Incidence of anterior uveitis in patients with axial spondyloarthritis treated with anti-TNF or anti-IL17A: a systematic review, a pairwise and network meta-analysis of randomized controlled trials
Source: Arthritis Res Ther. 2021 Jul 16;23:192. doi: 10.1186/s13075-021-02549-0 (PMC8283999; doi:10.1186/s13075-021-02549-0)

Additional File 4: Subgroup analysis

4A1: Subgroup analysis according to axSpA phenotype (AS only) – Anti-TNF mAb


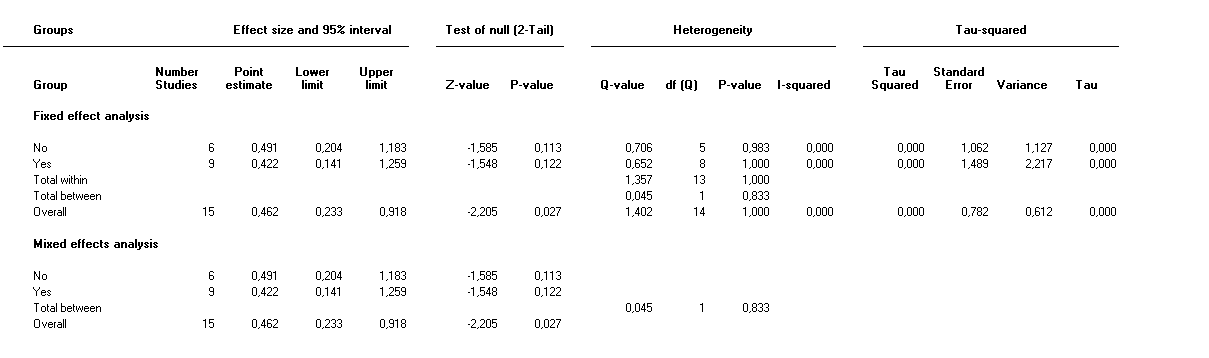


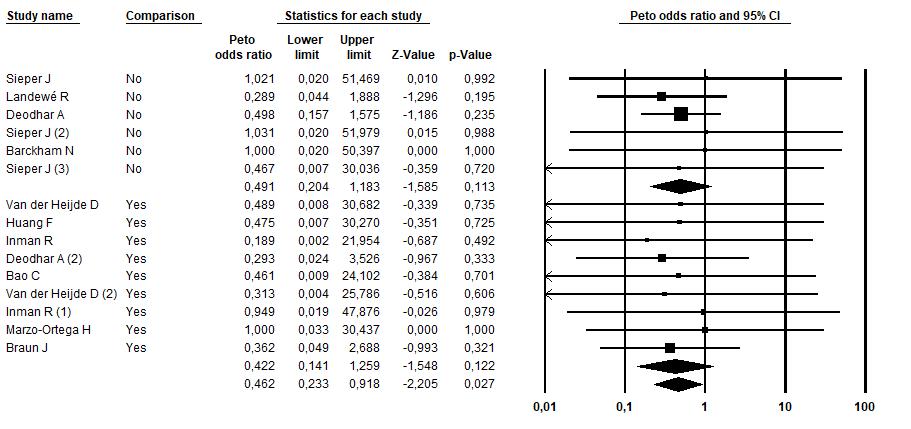


4A2: Subgroup analysis according to axSpA phenotype (AS only) – ETN


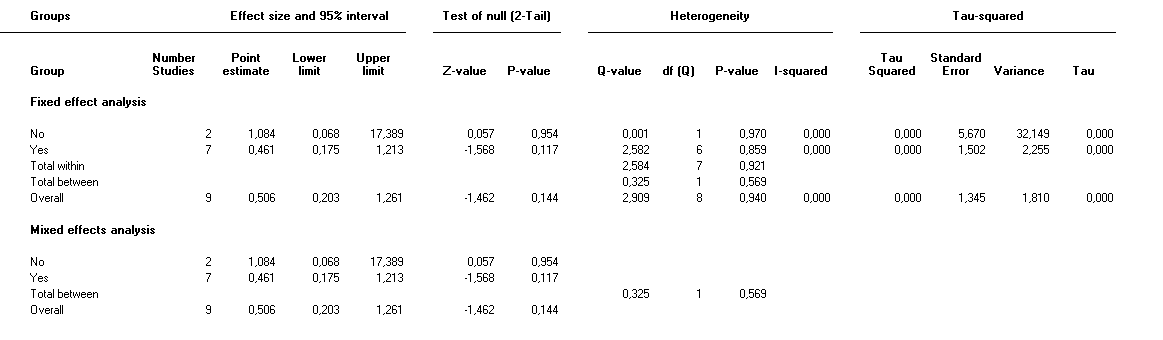


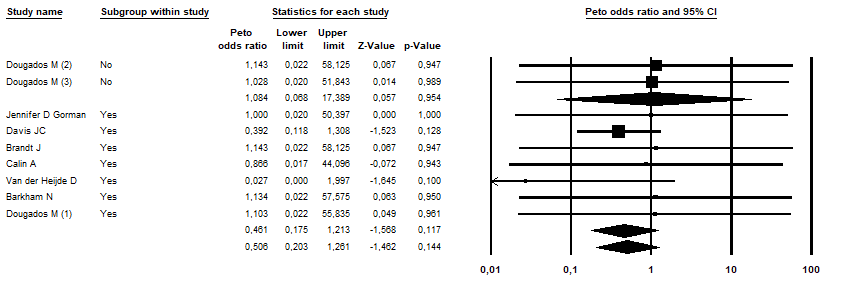


4A3: Subgroup analysis according to axSpA phenotype (AS only) – Anti-IL17A


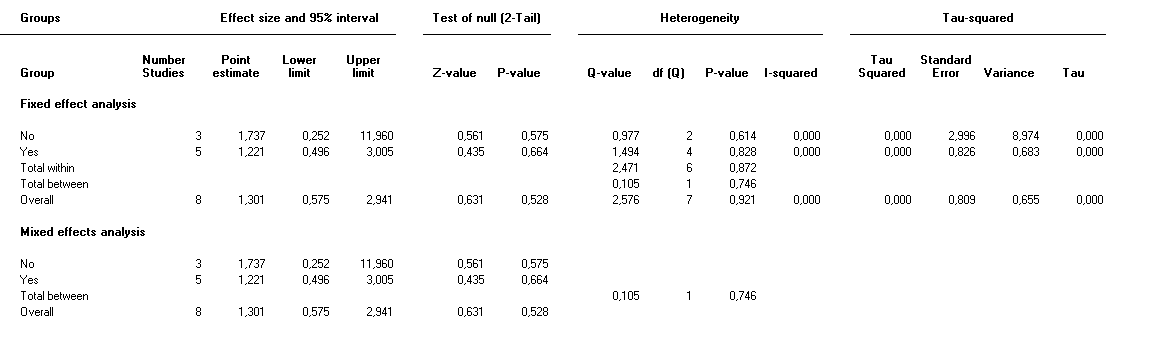


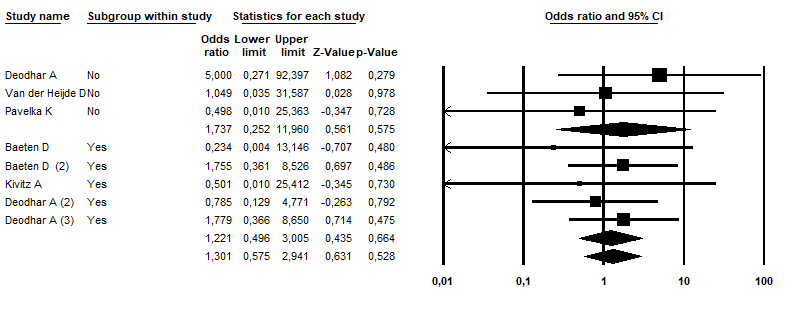


4B1: Subgroup analysis according to disease duration (<5 years) – Anti-TNF mAb


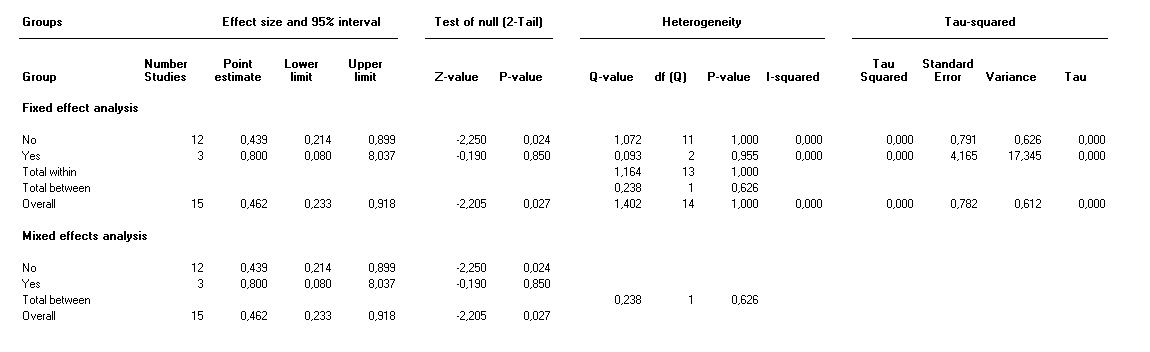


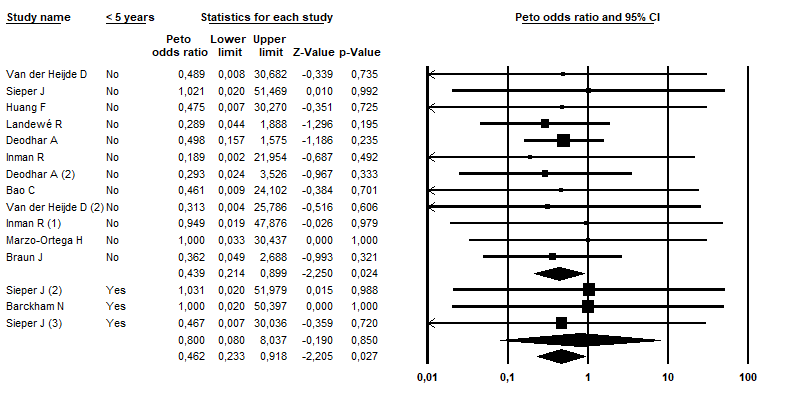


4B2: Subgroup analysis according to disease duration (<5 years) – ETN

Analysis not carried out as only one study was concerned

4B3: Subgroup analysis according to disease duration (<5 years) – Anti-IL17A

Analysis not carried out as no study was concerned

4C1: Subgroup analysis according to risk of biais (Cochrane RiOB) – Anti-TNF mAb


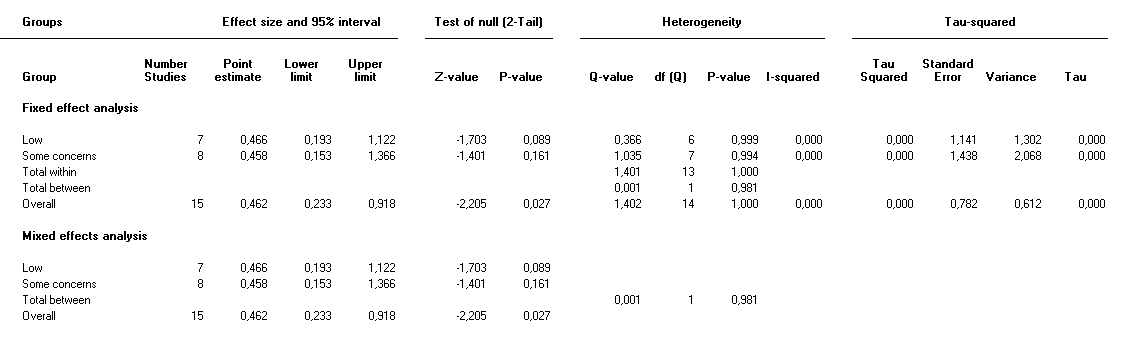

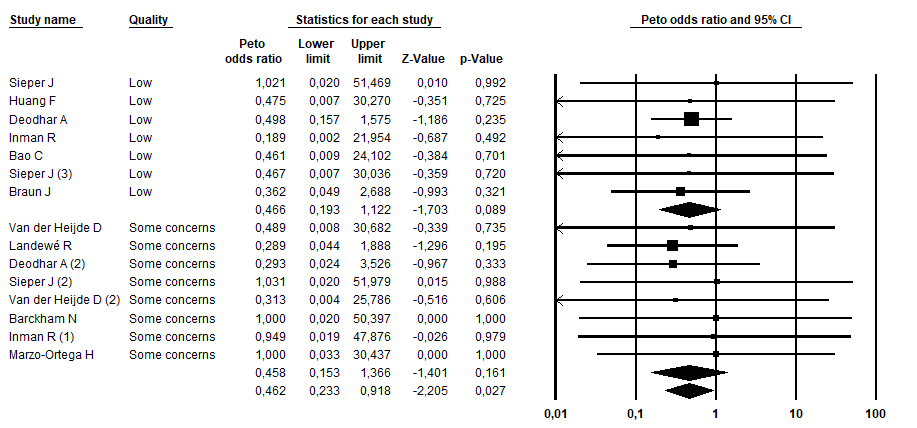


4C2: Subgroup analysis according to risk of biais (Cochrane RiOB) – ETN


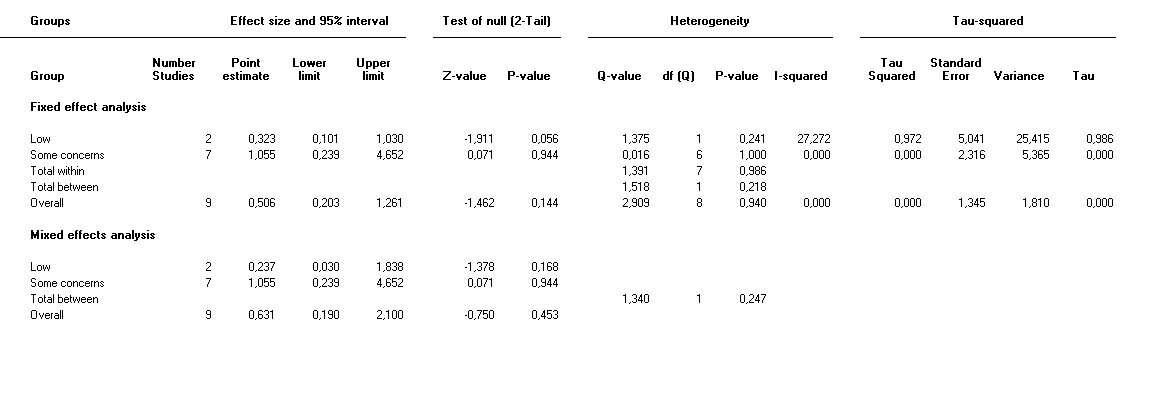


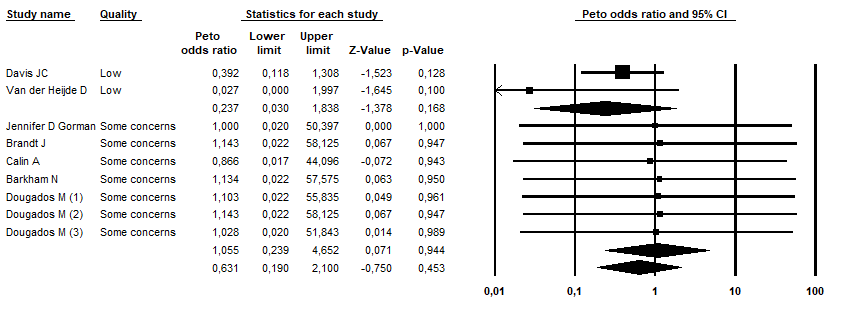


4C3: Subgroup analysis according to risk of biais (Cochrane RiOB) – Anti-IL17A

Analysis not carried out as only one study was classified as “some concerns”

4D1: Subgroup analysis according to history of AAU collected – Anti-TNF mAb


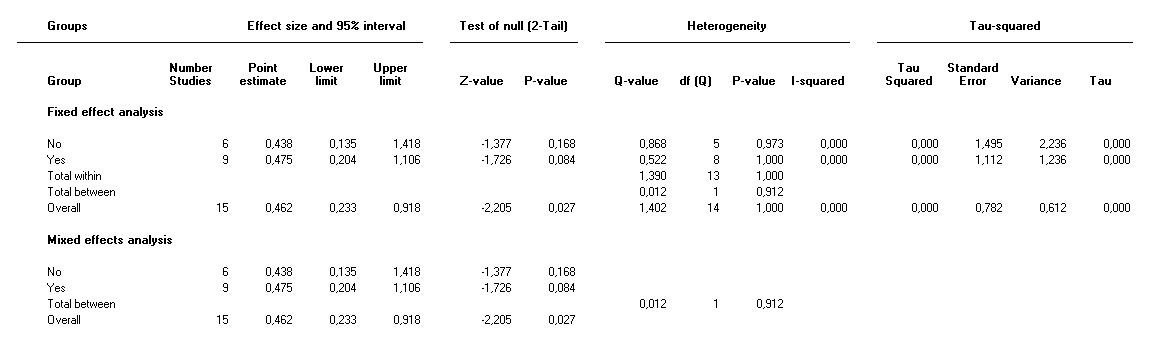


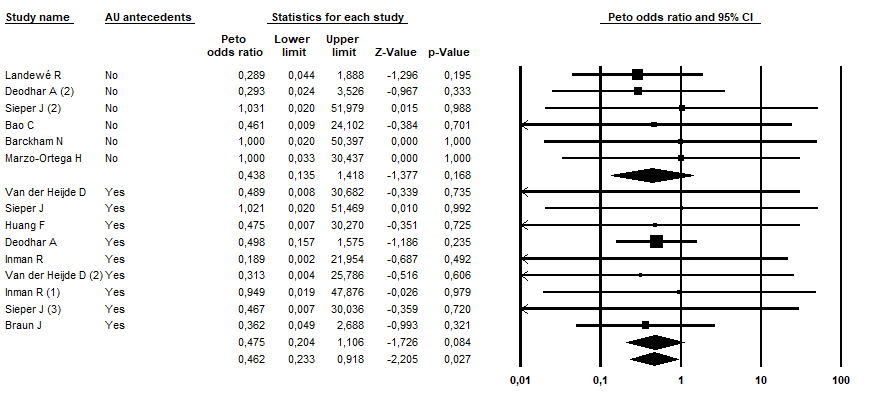


4D2: Subgroup analysis according to history of AAU collected – ETN


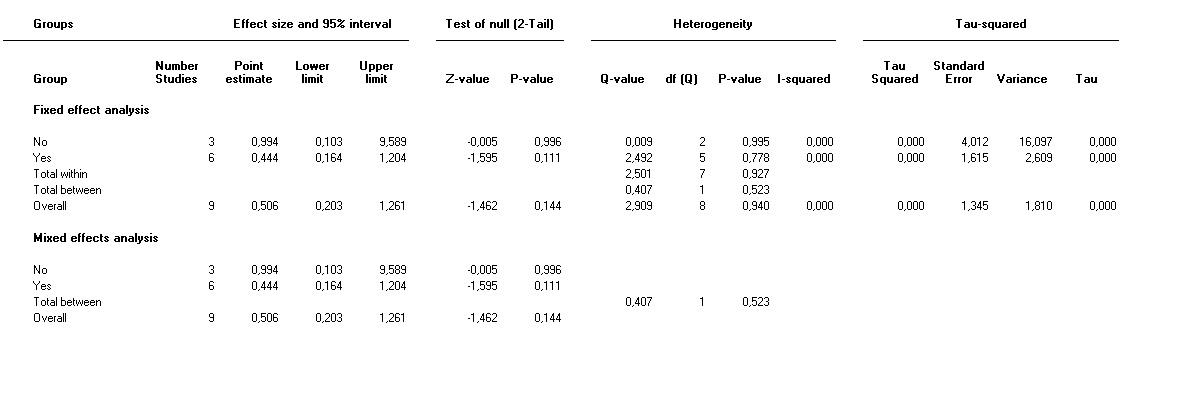


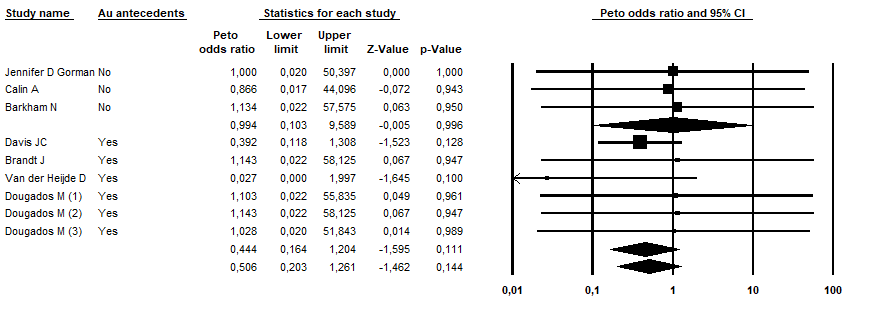


4D3: Subgroup analysis according to history of AAU collected – Anti-IL17A


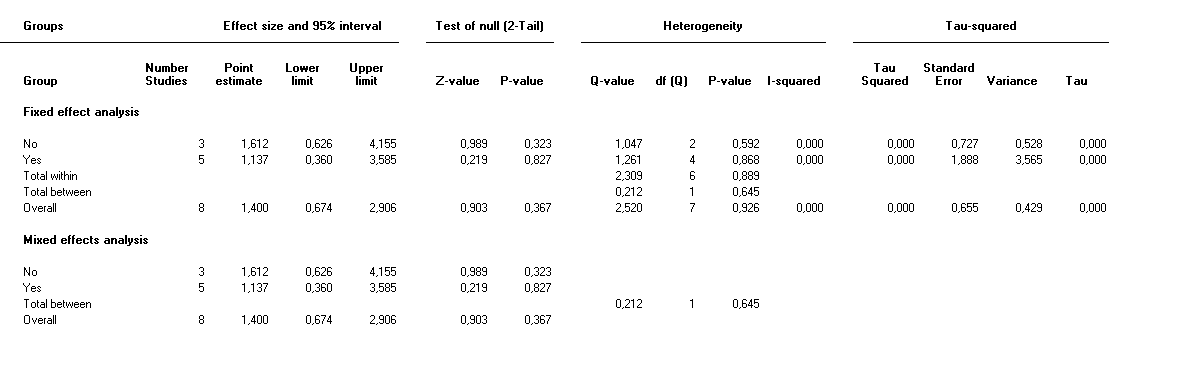


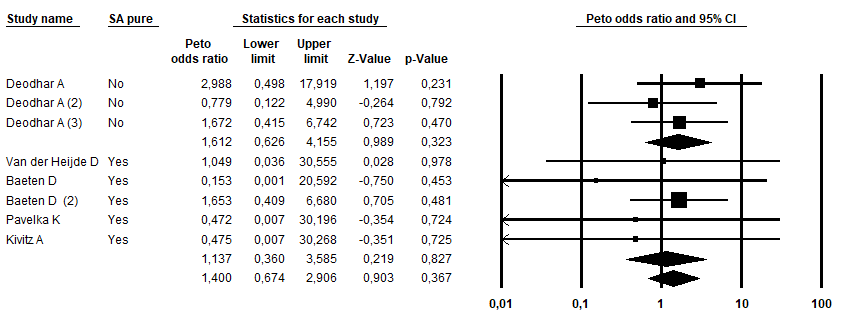

Supplement: Supplementary file 4 — Additional file 4. Subgroup analysis. [file 13075_2021_2549_MOESM4_ESM.docx]
